# Socio-ecological impacts of extreme weather events in two informal settlements in Nairobi, Kenya

**DOI:** 10.3389/fpubh.2024.1389054

**Published:** 2024-06-03

**Authors:** Anna K. Balakrishnan, Stephanie Otieno, Millicent Dzombo, LaNae Plaxico, Ebuka Ukoh, Lena Moraa Obara, Haley Brown, Christine Musyimi, Chloe Lincoln, Lyla Sunyoung Yang, Susan S. Witte, Samantha C. Winter

**Affiliations:** ^1^School of Social Work, Columbia University, New York, NY, United States; ^2^Columbia Global Center | Nairobi, Nairobi, Kenya; ^3^Rutgers, School of Social Work, The State University of New Jersey, New Brunswick, NJ, United States; ^4^African Mental Health Research and Training Foundation, Nairobi, Kenya

**Keywords:** health, mental health, women, climate, informal settlements, environmental justice, socio-ecological theory, Kenya

## Abstract

Climate change is expected to profoundly impact health and coping and widen social and environmental inequalities. People living in informal settlements are especially vulnerable to climate change as they are often located in ecologically sensitive areas more susceptible to extreme weather events (EWEs), such as floods, droughts, and heat waves. Women residing in informal settlements are especially vulnerable to climate change and related EWEs because they are more likely to experience worse health-related impacts than men but are less likely to have access to health-related services. Despite this inequality, there is a dearth of research that focuses on the impacts of EWEs on women in informal settlements. This study aims to explore the multidimensional impacts of EWEs on the daily lives of women in informal settlements through the lens of socio-ecological theory. Study data is from six monthly surveys (1 September 2022–28 February 2023) collected from a probability sample of 800 women living in two of the largest informal settlements in Nairobi, Kenya. This data is part of an ongoing longitudinal study that uses community participatory methods to investigate the effects of climate change on health and wellbeing in informal settlements by a team of 16 community health volunteers who lead data collection and provide expertise in ongoing analysis. Findings show profound impacts on women's health and wellbeing across individual, micro-, meso-, exo-, and macrosystems. These include physical and mental health, financial disruptions, property issues, social impacts, and impacts on their surrounding physical environment, such as disrupted food or water access, poor air quality, drainage issues, and safety concerns. In addition, findings highlight the critical importance of the chrono- and biosphere systems in research focused on the impacts of climate change and related EWEs among climate-vulnerable communities and marginalized populations within them.

## Introduction

Climate change is expected to profoundly impact health, mental health, and coping—widening social and environmental inequalities (1). People living in informal settlements—defined as areas lacking durable housing, access to basic infrastructure, and secure tenancy—are especially vulnerable to climate change as they are often located in ecologically sensitive areas that are more susceptible to extreme weather events (EWEs) such as floods, droughts, and heatwaves (2). Women who reside in informal settlements are especially vulnerable to climate change and related EWEs because they are more likely to experience worse health-related impacts than men (3–7), but less likely to have access to health-related services that have the potential to help them cope with negative health impacts resulting from EWEs (4, 8, 9). Despite this inequality, there is a dearth of research that focuses on the impacts of EWEs on women in informal settlements.

Home to approximately one billion people worldwide, informal settlements (also called slums) are often characterized by deficiencies due to limited provision of services to these communities, land tenure issues, government disinvestment, and social marginalization. They are broadly defined as areas that meet any of the five conditions: lack of clean water, lack of sanitation, non-durable housing, overcrowding, and insecure tenure [(10), p. 19]. In Kenya, specifically, the government has described informal settlements as “human settlement[s] characterized by dilapidated housing structures, overcrowding, abject poverty and unemployment, high insecurity incidences, insecure land tenure, exclusion of physical development, inadequate infrastructural services and often located in an unsustainable environment” [(11), p. iii]. In Nairobi, over half of the city's population (6.4 million residents in 2014) live in these rapidly expanding communities (12).

Climate projections for East Africa predict increased temperatures, heavier rainfall, and heightened flood and landslide risks in these areas (13, 14). A recent review article describes the myriad health impacts of climate change and EWEs in informal settlements, such as high mortality and injuries due to flooding and worsened sanitation and hygiene, as well as an increase in infectious diseases, skin problems, respiratory concerns, and vector-borne diseases. In Nairobi's largest informal settlement, Kibera, which is located along the Nairobi River and just upstream from the Nairobi Dam, frequent flooding has led to property damage, health issues, and economic disruptions (15). The combination of environmental challenges and socio-economic barriers residents face, such as social and political marginalization, poverty and inadequate infrastructure often rooted in legacies of government disinvestment (16), complicate climate adaptation and resilience efforts in these settlements (17).

Climate change is expected to exacerbate inequalities in health outcomes already disproportionately higher among women than men. Data suggest that women in informal settlements in Kenya already have the worst health outcomes of any population in the nation (18). One study found, for example, that prevalence rates of depressive disorder (17%), suicide attempts (13%), alcohol use (17%), and psychological distress (42%) in informal settlements in Nairobi are higher than national rates (19). Similarly, estimates of intimate partner violence (IPV) in informal settlements are very high, e.g., two-thirds of women experience past-year IPV (19) and 85% experience IPV at least once in their lifetime (20). Climate change exacerbates these health risks. For example, research suggests that in flooding or heat events, health risks are worse for women as they stay indoors due to insecurity and privacy concerns (21). Women in these settlements face a number of gender inequities such as disproportionate childcare responsibilities (22) and household water, sanitation, and hygiene duties that are more challenging to fulfill in the face of extreme weather. For example, ensuring the safety and wellbeing of children during a flooding event or accessing an adequate household water supply during a heatwave are challenging to fulfill in the face of extreme weather.

Women in informal settlements are not only more likely to experience worse impacts of climate change, but they have fewer coping resources. They are less likely to have access to healthcare and health insurance coverage compared to men (8). Women in informal settlements rely heavily on informal employment (2, 23, 24), limiting their access to employment-based health insurance and finances to pay fees for health services. They are also more disadvantaged with respect to living conditions, morbidity, mortality, and violence than men (18, 25). The impacts women in informal settlements experience due to EWEs associated with climate change have far-reaching ramifications across personal, social, ecological, and societal domains. To understand and address these impacts, examining the interaction between women in these settings and the contexts in which they live is essential. Bronfenbrenner's ecological systems theory has been widely applied in global health research to understand factors influencing health and wellbeing within and across multiple nested system levels: microsystem, mesosystem, exosystem, macrosystem, and chronosystem (26). More recently, ecological systems theory has been used to understand multi-system influences contributing to global health and mental health inequities and the impacts of climate change on the global economy and health (27). To our knowledge, no studies have employed this theory to understand the impacts of climate change on the health and wellbeing of residents in informal settlements. This paper uses socio-ecological theory to explore the impacts of EWEs in informal settlements in Nairobi, Kenya, through the shared experiences of women living there. It is our hope that this exploration will highlight women's experiences of EWE's multi-level impacts on their daily lives, and that this analysis will contribute to research to inform climate intervention and adaptation practices rooted in community members' lived experiences.

## Methods

Data were gathered from September 1, 2022 to February 28, 2023, as part of a longitudinal, quantitative investigation to examine associations and mediating effects of climate, mental health, and violence in informal settlements in Kenya. Monthly surveys were conducted at the household level with women residing in informal settlements in Nairobi. The results presented in this paper are derived from the initial six surveys out of the total 18 monthly surveys.

### Study site

**Figure 1:**
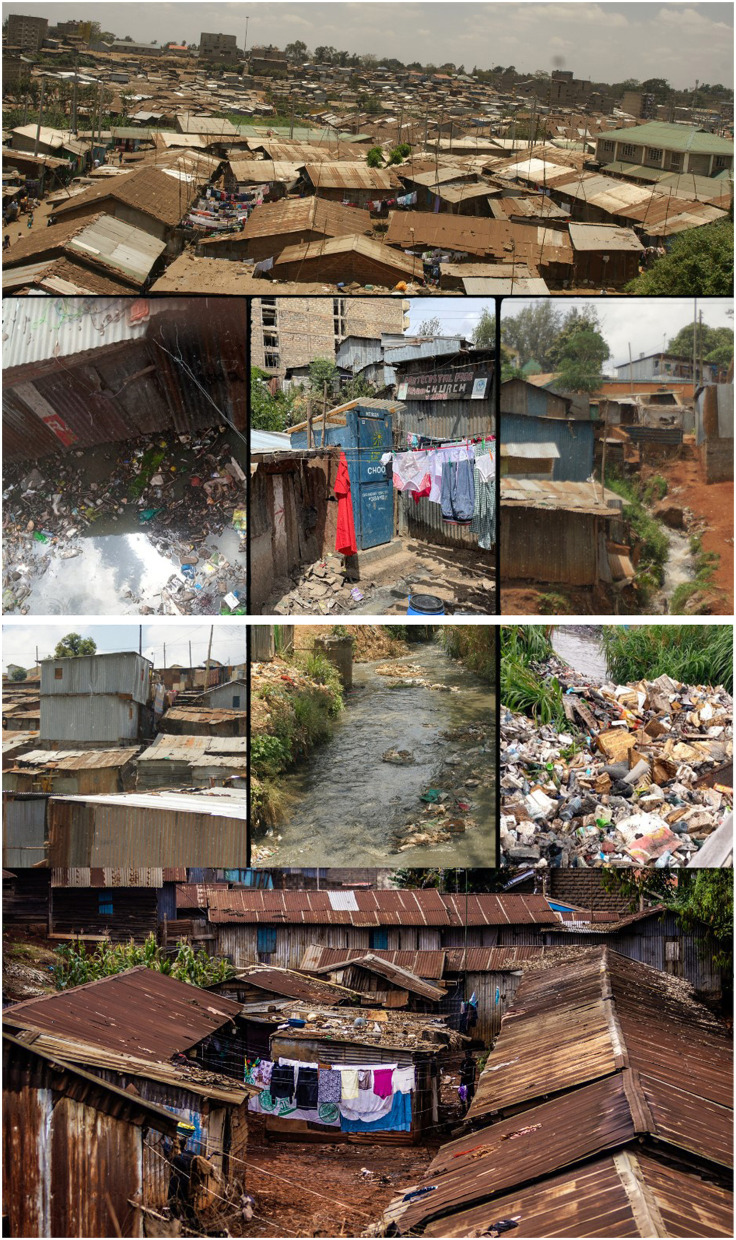
Images from Informal Settlements in Nairobi, Kenya.

**Figure 2:**
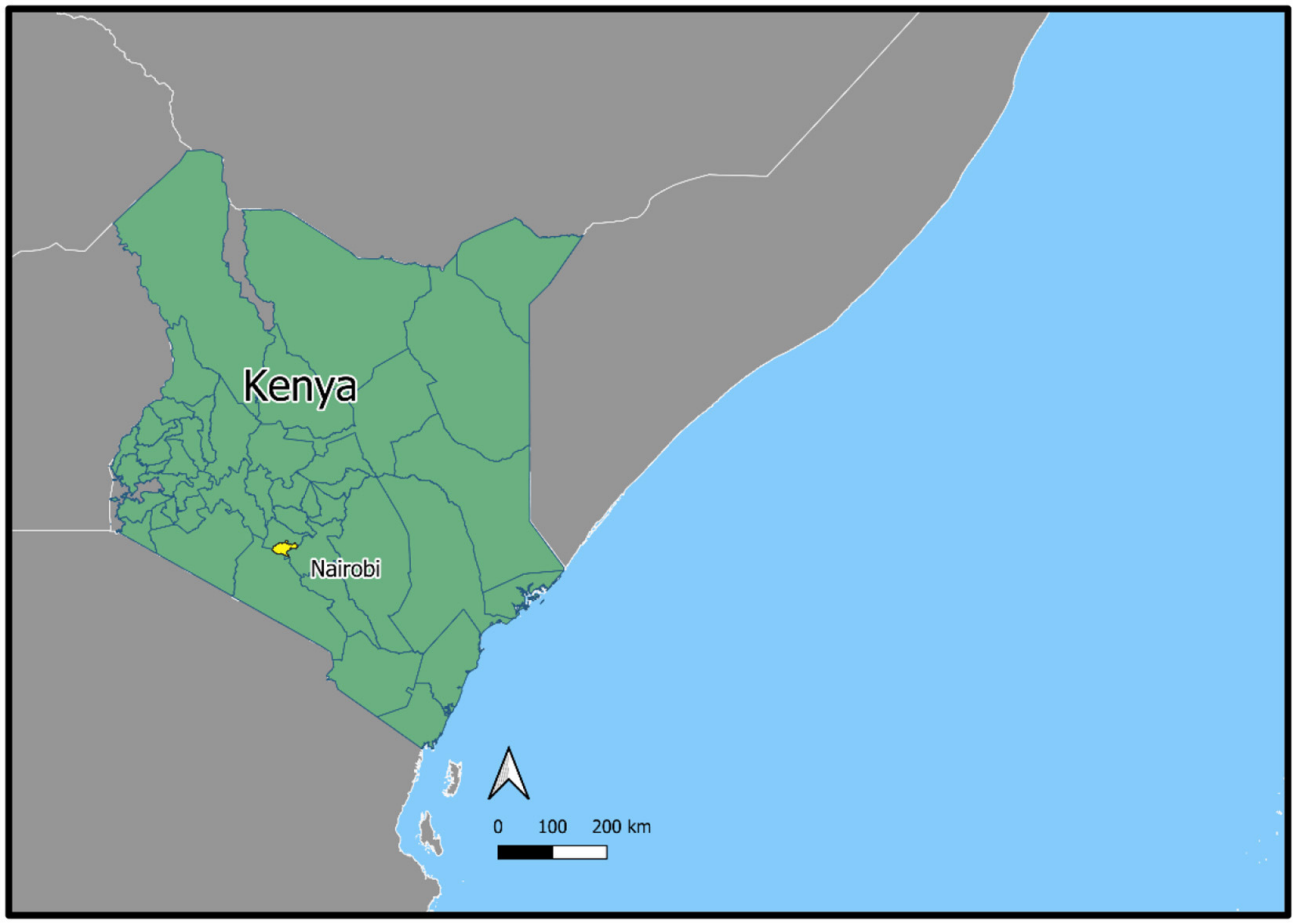
Map showing Nairobi's location in Kenya.

**Figure 3:**
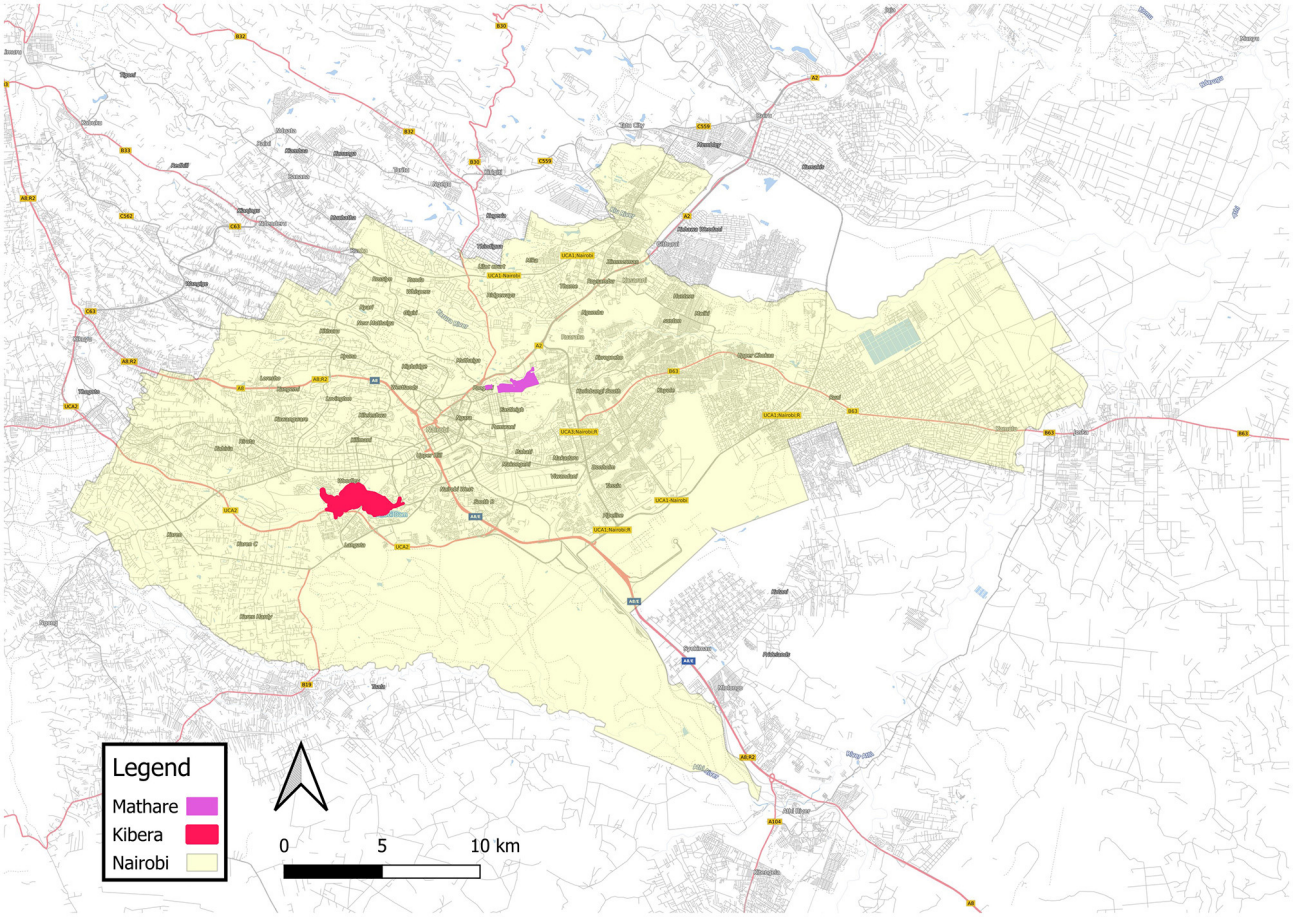
Map showing the location of informal settlements Kibera and Mathare in Nairobi. Source: OpenStreetMap and contributors (31).

**Figure 4:**
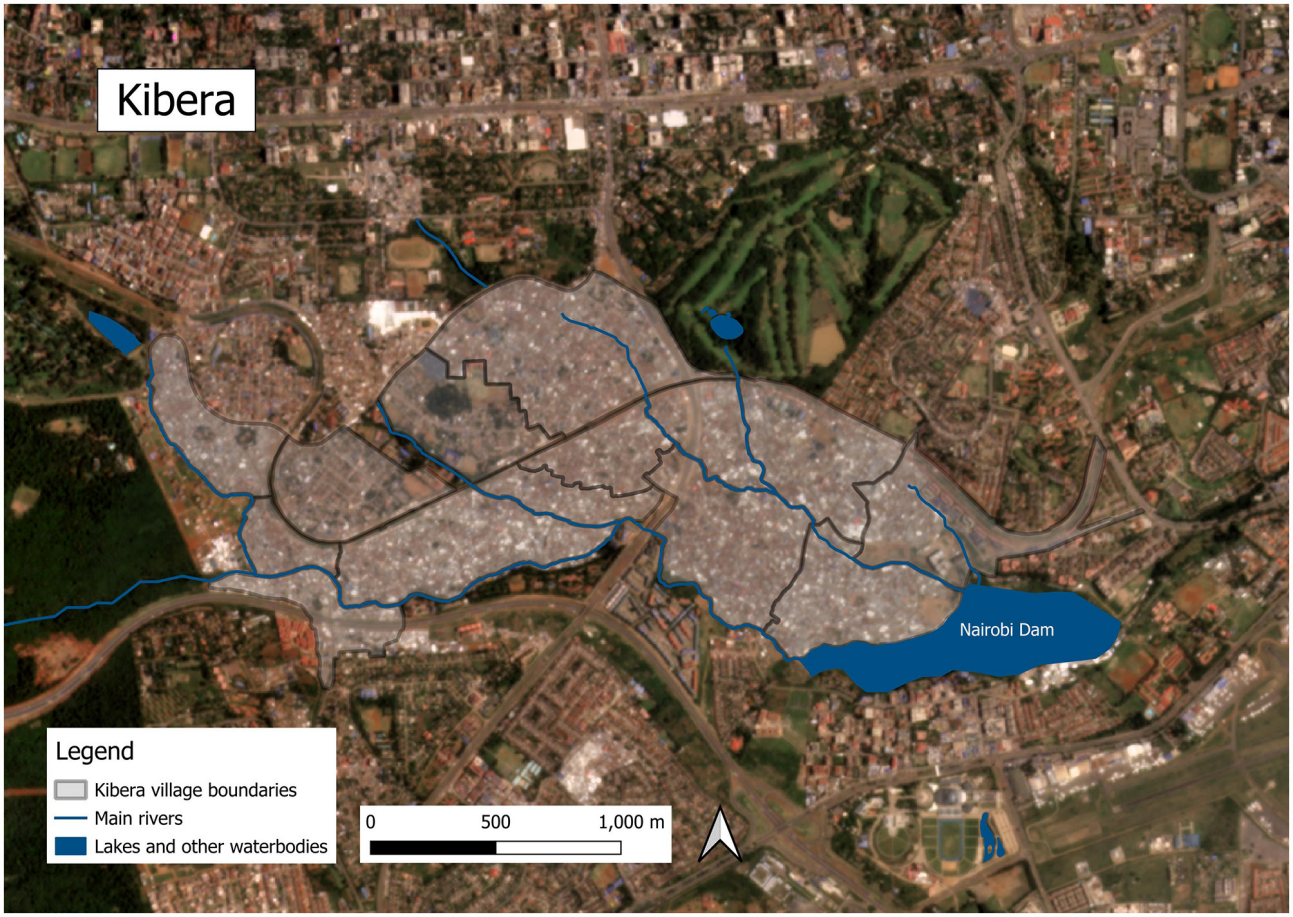
Map of Kibera and associated waterbodies. Satellite image © 2024 Planet Labs PBC (32). Waterbodies and river data from Humanitarian Data Exchange (HDX) (33).

**Figure 5:**
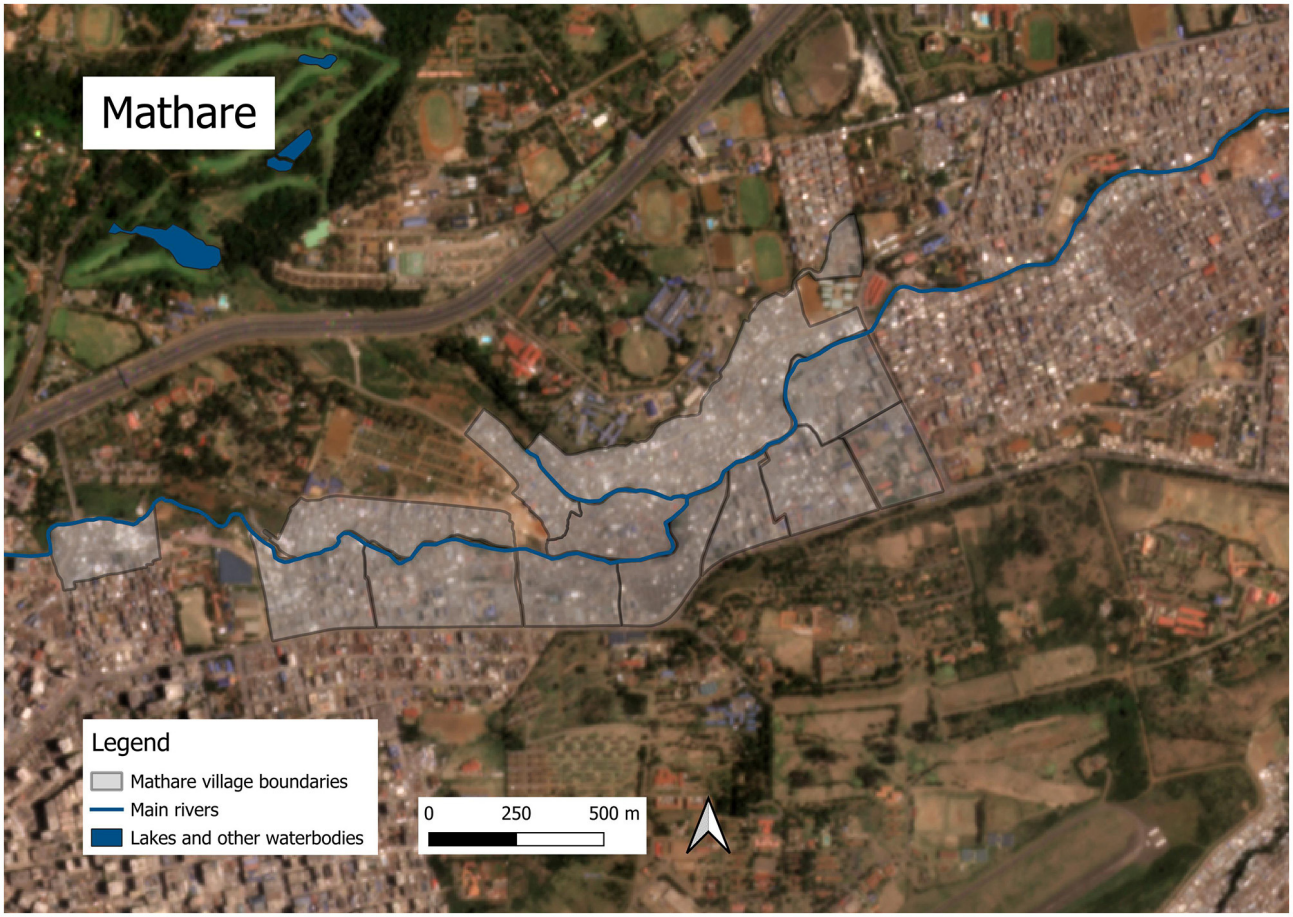
Map of Mathare and associated waterbodies. Satellite image © 2024 Planet Labs PBC (32). Waterbodies and river data from Humanitarian Data Exchange (HDX) (33).

Mathare and Kibera are two of Kenya's largest and most densely populated settlements. Systematic efforts to estimate the population of Kibera suggest that in 2010, the settlement was home to 200,000–250,000 residents (28) and Mathare had between 80,000–230,000 residents in 2010 (23). With a growth rate of around 5% per year (28), Kibera's population today would likely be around 350,000–400,000, and Mathare's around 84,000–250,000. Kibera comprises 14 villages (28); Mathare has 13 (23). In addition to high population density, informal settlements in Kenya are characterized by dense housing, with structures made of galvanized iron sheets, mud, wood, and concrete built very close to one another and narrow, unpaved paths between them with little vegetation–all factors that can exacerbate ecological sensitivity such as ambient temperatures (29). Kibera and Mathare are also situated in lowlands along Nairobi's major rivers, exposing residents to annual flooding and mudslides during heavy rains (13, 30). Please see Figure 1 for photos of Nairobi's informal settlements and Figures 2–5 for a map illustrating the geographic context of Mathare and Kibera.

Most residents in informal settlements in Nairobi, such as Mathare and Kibera, rely on informal economies for income, such as selling fruits and vegetables or mobile phone airtime and basic household necessities or casual labor “gigs” such as washing clothes and housework (2, 23). Access to water, sanitation, and electricity is inconsistent for informal settlements, with most residents relying on water from sources outside their homes/dwellings (18) and most relying on shared toilets outside their homes/dwellings (34).

### Study sample

The study team used a sampling approach that was previously implemented by the authors (19). The OpenStreetMap (.osm) platform was used for this research. In ArcGISPro version 3.0, a fishnet grid was superimposed on the OpenStreetMaps. Each grid cell measured 9 square meters (3 m × 3 m)—the approximate size of a mud or tin house or a room in a high-rise. A random selection function was used to identify 400 grid cells for sampling in each informal settlement area. Subsequently, GPS coordinates for 50 random grid cells were loaded onto each of the 16 community data collector's (CDCs) tablets. CDCs used Google Earth and Google Maps on their tablets to navigate to the nearest household corresponding to each GPS coordinate.

The quasi-random “last birthday” technique (35) was then used to identify one woman within each randomly selected household. Eligibility criteria required participants to be at least 18 years of age, proficient in either English or Swahili, and residents of the informal settlement for at least 6 months (i.e., not visitors or temporary residents). Swahili is the lingua franca in informal settlements. Using this method, the study team approached 863 households. Sixty-three women declined to participate, resulting in a total sample of 800 women in Kibera (*n* = 400) and Mathare (*n* = 400).

### Data collection

Household-level surveys were conducted monthly from September 1, 2022 to February 28, 2023 by 16 trained CDCs who are also women residents of Kibera (8) and Mathare (8). This data collection approach aligns with methods previously employed by the authors and in other studies (19). CDCs were trained on the principles of ethical research, quantitative data gathering, study protocols, and the World Health Organization's (WHO) ethical and safety recommendations for research concerning violence against women and sensitive topics (36). In adherence to these guidelines, researchers, local collaborators, and CDCs established safety protocols to be enacted if a participant reported instances of violence and/or adverse mental health outcomes. All participants provided written consent following the informed consent process, during which CDCs provided study information and participants were given opportunities to ask questions about the study and their participation. The 60–90 min survey was conducted in participants' homes and administered using tablets.

During each survey, participants were asked about whether they or members of their household experienced any EWE(s)—defined as “an event that is rare at a particular place and time of year” (37)—in their settlement in the past month. If they said “yes,” they were then prompted to specify the type(s) of the event(s) and the corresponding date(s) of event(s). Additionally, women were presented with a roster of typical EWEs (extreme heat, extreme cold, flood, landslide, heavy downpour, drought). They were asked whether they or their household had encountered any of those events, and again, if so, they were prompted to specify the date(s). Respondents were also asked to identify any other EWE that was not listed but had occurred in the past month. To capture the impacts of EWEs, participants who reported that they or members of their household had experienced an EWE were asked whether and how the EWE(s) affected their daily lives or the lives of other members. Responses were transcribed verbatim.

### Analysis strategy

We employed Bronfenbrenner's ecological systems theory to guide the qualitative coding of women's reported impacts from EWEs within a socio-ecological systems framework. Five research team members coded and categorized women's verbatim descriptions of the EWEs' impacts on daily life as part of our thematic analysis process (38). To start, two team members independently coded the verbatim responses collected during the baseline survey, which led to creating a working codebook. Three coders then used the codebook to systematically code the monthly follow-up surveys (*n* = 5) from October 2022 to February 2023. A fourth coder reviewed the codes separately to confirm their congruency and accuracy. Any discrepancies in coding and necessary codebook adjustments were addressed collaboratively with the entire research team. Impacts were categorized into thematic areas (financial, physical health, environmental health) and separated into distinct socio-ecological system levels. Codes were created based on direct participant reports of impacts. Responses that did not fit into an existing system were noted, and adjustments to the theory are explored in the results and discussion of this paper. Upon the research team's endorsement of the finalized codes, a frequency analysis of the impacts of EWEs was conducted.

## Results

### Demographics

The average age of participants enrolled in this study was 47 years, but participants' ages ranged from 19 to 80. Most participants (47%) completed primary school, 26% completed secondary education, and just over 1% attained higher education. Less than 5% had no formal education. In terms of community tenure, 64% of participants had resided in the settlement for over 10 years, with 34% living there for 1–10 years and < 2% for less than a year. About 55% were married, 9% were widowed, and 10% were divorced, while 17% were single, and 9% were in relationships but not married. Work patterns varied, with ~43% working or engaging in income-generating activities daily or almost daily and 22% not working. Nearly half (46%) identified as household heads, with an average household size of 4.5 individuals and a maximum of 17. Most participants had at least one child, as 44% had 1–2 children, 36% had 3–4 children, and 14% had more than five children; 6% were not parents. About half (53%) of participants' partners were employed.

### Impacts on women living in informal settlements in Nairobi within adjusted ecological systems model

**Figure 6:**
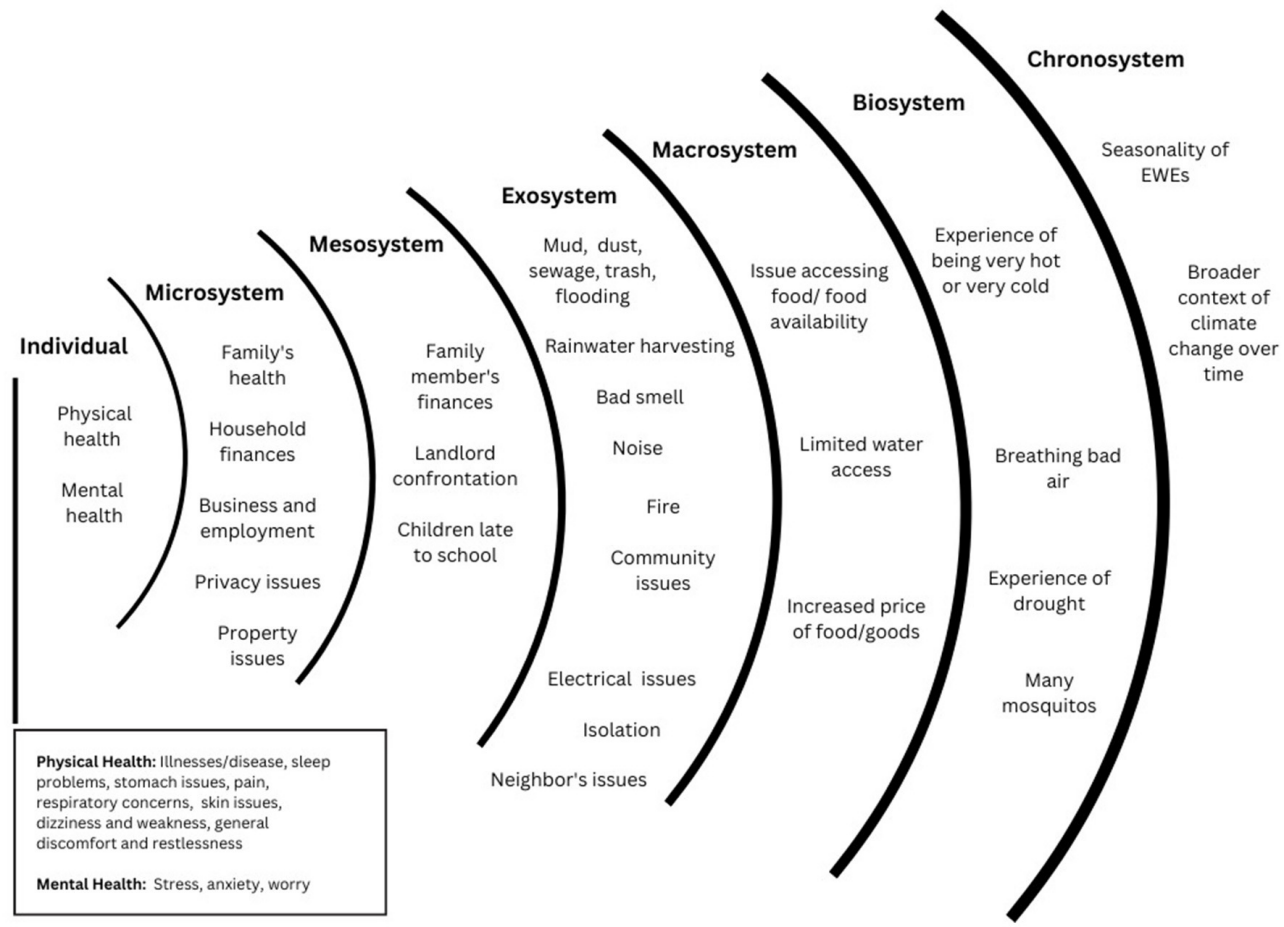
Impacts of EWEs on Women Living in Informal Settlements in Nairobi within Adjusted Ecological Systems Model.

Participants described a range of impacts relating to their health and wellbeing across the socio-ecological levels, including physical and mental health, financial wellbeing, property concerns, physical environment, and economic influences. We introduced an additional level to the original socio-ecological framework, the biosystem, to capture participants' responses describing their experience of EWEs as an impact itself and pointing to humanity's direct relationship with nature and the broader environment/climate in which we are embedded. While not directly captured in participants' responses, the chronosystem level also emerged in the data structure. Please see Figure 6 for details.

### Individual-level impacts

At the center of Bronfenbrenner's ecological model is the individual. Participants described personal physical health impacts from EWEs, including increased illnesses from the common cold (primarily due to colder temperatures than usual) to malaria (due to increased mosquitos in the region). Participants reported sleep disturbances and fatigue across multiple EWEs, including flooding, higher-than-usual temperatures, downpours, and increased mosquitos. It is important to note that while increased mosquito population is likely a consequence of other EWEs, this phenomenon was named as an extreme weather event by women in this study. Therefore, it is included as an EWE in honor of the women's definitions. Participants experienced stomach issues (stomachache, difficulty eating) during bad air events and respiratory concerns during higher-than-usual, colder-than-usual temperatures, extreme wind, and bad air quality events. Additional physical health impacts include chest pain and tightness, high blood pressure, pain, skin issues (i.e., rashes, many bites), general discomfort and restlessness, difficulty walking, dizziness, and, less frequently, pneumonia and cholera. One participant described how hot weather heightens the effect of their marijuana use, which helps them to sleep better. Participants described mental health impacts as a result of EWEs, namely worry, stress, and anxiety. See Table 1, “Individual level impact frequencies,” for details.

**Table 1:** Individual level impact frequencies.

**Code**	**Flood**	**High temps**	**Cold temps**	**Drought**	**Downpour**	**Wind**	**Bad air**	**Mosquitos**	**Totals**
Health impacts									933
Cold or flu		21	51		7	34	6		119
Sleep issue	1	50			23			40	114
Fatigue		86	3						89
Malaria		3	9		3			67	82
Stomach issue			1		1		60		62
Headache		37	2	1	1				41
Illness/disease		9	10	1	7	1	3	9	40
Respiratory		17	9			5	5		36
Eating issue				3	1		31		35
Discomfort		21	3				9		33
Skin issue		23	2		1			4	30
Bites								30	30
Overheating		29							29
General hangindent7pt sickness/not hangindent7pt feeling well	1	10	9		3		4	1	28
Restlessness		21	4						25
Allergy	2	9	5		1	2	1		20
Difficulty hangindent7pt walking		19	1						20
Chest problem			14		2				16
Congestion		2	9		3				14
Pain		3	10		1				14
Weakness		9	2		1				12
Fever		9	2						11
Dizziness		9							9
Nosebleeds		9							9
Asthma		2	4						6
Pressure		3							3
Cholera		1				1			2
Pneumonia			2						2
Diarrhea			1						1
Drugs		1							1
Mental health impacts									49
Stress, hangindent7pt anxiety, worry	1	22	5	10	8	1	1	1	49

### Microsystem-level impacts

Impacts in the microsystem include those that relate to the individual's immediate surroundings. The microsystem is considered the innermost, nested system of the socio-ecological framework. Women reported that EWEs impacted the health of their children or other family members. Participants described financial impacts, including disruptions to their employment or business and increased household costs, which impact the financial wellbeing of the entire family/household. Participants reported a disruption to employment or business due to flooding, higher-than-usual or colder-than-usual temperatures, drought, downpours, extreme wind, and bad air quality. Household costs increased by purchasing additional items to adapt to EWEs, including cool-weather clothing, food, and coal for heating. Participants described a general financial concern, i.e., insufficient money to pay for their needs. In rare cases, participants described how the EWE improved their financial wellbeing by improving their business or employment or decreasing costs, e.g., clothes dry faster in higher-than-usual temperatures—meaning that women who wash others' clothes for a living can take on more jobs in a day/week.

Participants reported property issues because of EWEs. Flooding caused water, sewage, trash, or mud to enter homes. Participants described damage to household structures due to inadequate house materials, such as roofing materials (especially iron sheets) or walls leaking or blowing away, primarily during downpours and wind events, respectively. Participants also reported damaged personal belongings due to EWEs, especially flooding and heavy downpour events. Finally, participants reported bad smells in their homes during bad air quality events and fire and electrical issues during downpours. While not explicitly described by participants, property concerns are likely connected with a financial impact as women and their families seek to repair home damages. Additionally, social impacts included a lack of privacy during heat events because most participants navigate shared spaces with their families while trying to stay cool. See Table 2, “Microsystem impact frequencies,” for details.

**Table 2:** Microsystem impact frequencies.

**Code**	**Flood**	**High temps**	**Cold temps**	**Drought**	**Downpour**	**Wind**	**Bad air**	**Mosquitos**	**Totals**
Health impacts									80
Children's health		20	19	2	6	3	2	18	70
Family member's hangindent7pt health		1	3			2	1	3	10
Financial impacts									266
Business or hangindent7pt employment disrupted	12	59	24	16	56	12	2		181
Not enough money		4		40					44
Increased costs		7	3	9	8				27
Business or hangindent7pt employment improved		6			2				8
Daily duties hangindent7pt disrupted		4							4
Decreased costs		1		1					2
Property issues									537
Flooding/water in hangindent7pt house or property	41	1			260				302
House materials hangindent7pt inadequate	3	18	4		93	24			142
Belongings damaged	4				17	10			31
Sewage	5	1		1	7	1	2		17
Muddy/dirty/dusty	1					14			15
Trash entering hangindent7pt house or property	2				3	6			11
Displacement	3				5	1			9
No mosquito net								4	4
Bad smell		1					2		3
Fire					2				2
Electricity					1				1
Social impacts									1
Privacy issue		1							1

### Mesosystem-level impacts

EWE impacts classified in the mesosystem include those interactions between the participants' immediate surroundings (i.e., their family members) and the larger community. Several EWEs impacted participants' family members' finances and work lives by disrupting their business or employment. One participant shared that a downpour affected them by making children late for school. As a result of property issues, participants also reported having issues with their landlords while trying to navigate who was responsible for paying for home repairs. See Table 3, “Mesosystem impact frequencies,” for details.

**Table 3:** Mesosystem impact frequencies.

**Code**	**Flood**	**High temps**	**Cold temps**	**Drought**	**Downpour**	**Wind**	**Bad air**	**Mosquitos**	**Totals**
Financial impacts									4
Family hangindent7pt financials		1		1	2				4
Social impacts									2
Landlord hangindent7pt confrontation						2			2
Physical environment									1
Children late to hangindent7pt school					1				1

### Exosystem-level impacts

Exosystem impacts relate to the participant's interactions with the larger community. Participants reported various issues related to their physical environment, including poor drainage and excess water or flooding, mud, dirt, dust, and trash in the community. As a potential positive impact on individuals and families, downpour events resulted in increased rainwater harvesting in the community. It should be noted that increased rainwater harvesting may positively and negatively affect different individuals and the community. Rainwater harvesting decreases the cost of buying water for individual families but may decrease business for women who earn their living by selling water. Additionally, the quality of water from rainwater harvesting is variable. Since there is often no formal trash collection system, some people dispose of trash or feces on rooftops, which could affect water quality from harvesting. Women also reported an impact on clothing in two ways. First, EWEs affect the rate at which clothes dry. Many women earn their living by washing clothes for others. When the clothes drying rate changes, this impacts how much clothing they can wash/dry in a day or week (more during higher-than-usual temperature events and less during cold events). Women also suggested that the rate of clothes drying can create conflict with neighbors or other community members. For example, when there is limited shared space designated for drying clothes (lines), colder-than-usual temperatures can delay drying and create space challenges. Additionally, temperature events, especially, can generate wardrobe issues—women having to buy additional clothes (cold events) or deal with privacy issues when removing clothes (heat events).

Participants describe social impacts at the community level, including isolation due to EWEs that affect participants' ability to leave their homes and participate in society (flooding, colder temperatures than usual, downpours). In addition, participants shared the impact of EWEs on their neighbors' lives (i.e., community fires or flooding cause neighbor displacement as well as their own). See Table 4, “Exosystem impact frequencies,” for details.

**Table 4:** Exosystem impact frequencies.

**Code**	**Flood**	**High temps**	**Cold temps**	**Drought**	**Downpour**	**Wind**	**Bad air**	**Mosquitos**	**Totals**
Physical environment									266
Muddy/dirty/dusty	2	22		3	8	45			80
Clothes (drying hangindent7pt or wardrobe issue)		6	41		1	1			49
Drainage issue	8	2		2	24		4	1	41
Rainwater hangindent7pt harvesting	1				29				30
Bad smell		1		8	1		16		26
Sewage	3	1		6	3		7		20
Trash	1			1	6	2			10
Fire				1	2		1		4
Community hangindent7pt issue					3				3
Noise						2			2
Electricity issue					1				1
Social impact									15
Isolation	1	3	1		2				7
Neighbors	4				3				7
General					1				1

### Macrosystem-level impacts

Macrosystem impacts include broader societal-level influences. Participants reported food and water shortages during drought and high temperatures. Participants' reports of overall rising prices of food and goods were closely related to these shortages. These shortages and subsequent cost increases stem from supply and demand issues during droughts (livestock dying, loss of crops) or downpours (damage to produce). See Table 5, “macrosystem impact frequencies,” for details.

**Table 5:** Macrosystem impact frequencies.

**Code**	**Flood**	**High temps**	**Cold temps**	**Drought**	**Downpour**	**Wind**	**Bad air**	**Mosquitos**	**Totals**
Physical environment									326
Issue accessing hangindent7pt food/food availability		23	1	147	3	3			177
Water access		65	3	75	6				149
Financial impacts									121
Increased price of hangindent7pt food/goods		21	1	92	6	1			121

### Biosystem-level impacts

The biosphere refers to the global ecological system that considers humans as embedded beings within the earth system: nature impacts us, and we impact nature (39, 40). Bronfenbrenner's ecological systems theory is a profoundly anthropocentric model—ignoring consideration of human-nature interconnections, i.e., how human beings are embedded within and are greatly affected by and greatly affect climate and the broader physical/natural environment. While the biosystem is not traditionally considered in most socio-ecological analyses, our findings suggest it is critical to understanding women's descriptions of the EWE impacts they experience. As an example, many participants described the impact of the event as the same as the event itself, e.g., the impact of an extreme heat event is being “very hot,” the impact of an extreme cold event is being “very cold,” the impact of a bad air quality event is “bad air quality,” or the impact of a mosquito event is “too many mosquitos.”

See Table 6, “Biosystem impact frequencies,” for details.

**Table 6:** Biosystem impact frequencies.

**Code**	**Flood**	**High temps**	**Cold temps**	**Drought**	**Downpour**	**Wind**	**Bad air**	**Mosquitos**	**Totals**
Physical environment									224
Very hot		128	4	1				1	134
Very cold	1	2	31	1	9		1		45
Bad air							15		15
Drought		1							1
Flooding/water hangindent7pt everywhere	14			2	1				17
Mosquitos					1			11	12

### Chronosystem-level impacts

According to Bronfenbrenner's adjusted ecological systems theory, chronosystem-level impacts include those related to time (26). In this study, the timing and seasonality of EWEs is an important consideration. The very definition of EWE as “an event that is rare at a particular place and time of year” (37) requires consideration of the timing of an event. The nature of the study positions these findings within the temporal context as women determined whether an event was extreme. Women made these determinations within the context of having experienced Kenya's typical seasonal changes for at least the past 18 years (the minimum age of participation). While some of the EWEs women reported experiencing fall close to or within the range of dates that align with Kenya's historical seasons (hot/dry season—December-February; rainy season/long rains—March-April; cold season—June-August; and short rains—October-November), the quality or timing of the events were experienced as extreme despite the seasonality of some events.

## Discussion

Study findings contribute to a more comprehensive understanding of the impacts brought by climate change-related EWEs on women's health and wellbeing from the perspective of women living in informal settlements. Findings corroborate many physical and mental impacts documented in existing literature focused on other populations while introducing some new health-related impacts and key environmental, economic, and social impacts. Additionally, findings reveal the complex, co-occurring, and interconnected nature of the impacts of climate change related to EWEs for women living in climate-vulnerable communities. Finally, findings highlight the critical importance of the chrono- and biosphere systems in research focused on the impacts of climate change and related EWEs among climate-vulnerable communities and marginalized populations within them. We discuss these findings in more detail and identify areas for future exploration, intervention, and adaptation for one of the communities/populations expected to be most vulnerable to climate change.

Findings from this study complement existing research on the negative physical health and mental health outcomes associated with climate change (3, 4, 6, 41–43). Physical health impacts previously explored by researchers in the context of informal settlements include a range of health outcomes including mortality, infectious diseases, injury, illness including fevers, cold and flu, rashes and skin infections, and sleep issues [(44, 45); please see Hambrecht et al. (21) for review article]. These health impacts are of particular concern when taking into account that women living in informal settlements experience compounding barriers to their health and wellbeing, such as worse mental health outcomes, high rates of intimate partner violence, and less access to healthcare and health insurance (8, 19, 20), which are further exacerbated by EWEs along with impacts on livelihood, coping, and adaptive capacity that indirectly affect health (21). The negative health effects experienced by women in informal settlements, when viewed in light of the economic and social hardships they endure, should be a priority for researchers, policymakers and development communities. These groups should focus on mitigating the consequences of climate change on these women's health and living conditions.

Women's descriptions of impacts on their property and physical environment provide a vivid picture of the vulnerability of living in the unique physical, political, and built environment in informal settlements in the face of climate change and related EWEs. Informal settlements are characterized by a lack of essential services like clean water and sanitation, non-durable construction, overcrowding, and insecure tenure [(10), p. 19] as well as social and political marginalization and government disinvestment (2) that contribute to the climate vulnerability of these communities. Examples of political marginalization include exclusion from formal services and markets, impunity for officials who fail to act or act violently or immorally, and inadequate security and legal protection (46, 47). These aspects of the physical, political, and built environment, combined with the ecological sensitivity of many settlements (2), have serious implications for the health and wellbeing of residents (48, 49). Women named impacts to the physical environment, including flooding, trash, dirt, mud, mosquitos, bad air quality, and sewage in the community, along with electrical issues, fires, and other community-level issues. These issues are particularly pronounced in informal settlement communities where there is exclusion from formal solid waste collection (50, 51) and other essential services like water, sanitation, and electricity (2). Women described their vulnerability to the impacts of EWEs, including their roofs or walls, made chiefly of recycled corrugated iron sheets, being ripped/blown off of houses during extreme wind events. Given extensive challenges with land tenure, threats of eviction, and limited resources available for development in informal settlements, many residents and landlords do not invest in permanent housing structures (52). Consequently, housing materials are often insufficient to prevent rainwater from pouring into homes during heavy downpours and prevent water, sewage, trash, and mud from entering the home during flooding. Relatedly, women face damage to their belongings and businesses and even potential displacement from the home. During periods of drought and higher-than-usual temperatures, women describe high levels of dust and dirt that impact the cleanliness of their homes, their ability to sell food (especially along roads/areas with heavy foot traffic), and the quality of the air they breathe. This is likely exacerbated by the proximal siting of factories, roads, railways, dumps, and industry to settlements (53) as well as the limited vegetation and system of exposed earth areas/pathways and community spaces in these communities (29). These impacts present concerns of environmental health and safety as well as health impacts.

Our findings show that the financial impact of EWEs is experienced at almost every level of women's ecology. At the household/familial and even community (exosystem)-level, women experience disruptions to employment and businesses and increased costs for essential goods such as food and water (especially during rationing). For example, at the household/familial and community level, changes to the physical environment, such as heavy rains, flooding, extreme heat or cold, or dust, can limit women's customer base or reduce women's ability to leave their homes to work or run their businesses. Some women described street vendors losing customers when it is too dusty (from heat events or droughts) because people do not want to risk buying foods, especially prepared/cooked/ready-made, that are covered in dust. These findings complement existing research that notes the impacts of EWEs on job disturbances and loss of income in informal settlements [see Hambrecht et al. (21) for narrative review]. At a macrosystem level, rising prices of food and other goods, especially in response to drought or floods at the national level, impact women's daily lives. Increases in prices at the macro- and/or community levels and/or lack of availability of stock (e.g., from lost livestock or crops due to EWEs at the country level) limit the availability of products/goods women can afford to buy and/or sell. These impacts are especially alarming when considering the preexisting financial context of many residents living in informal settlements, where many households live at or below the universal poverty line of US $2.15 per day (9, 54). Households' expenses often exceed their monthly income (55), as they pay for rent, food, school fees, shared toilets and showers, water purchasing, and transportation costs (56). For example, in Mathare, researchers have found that households typically face an average monthly deficit of KES 3000 (US $30) (23). Indeed, EWEs' financial impact on residents of informal settlements exacerbates already serious financial and labor market inequalities faced by residents of informal settlements.

It is important to note that the research team only coded a response to financial impact if women explicitly named it as such (e.g., “I wasn't able to go to work” or “I had to pay more money for food”), which are reflected in the frequency tables. However, impacts on multiple system levels can impact women's finances, such as paying for repairs due to property issues or for health concerns of self/other family members that require medical/specialist attention. As women care for themselves, their families, and their households, they have to make daily decisions about allocating funds and navigating shocks to their finances, which present additional strain. Of note, however, there are some cases where women described improvements to their lives due to EWEs, such as business picking up in dry seasons for women who are washing clothes or women being able to buy raw products, e.g., fruits and vegetables, as a result of advantageous weather, that they can sell at a markup or use to make and sell prepared foods.

Amidst the wide range of challenges for those living in informal settlement communities, research has found robust systems of care and social support: community members help to take care of one another (57, 58). Of note, women expressed great concern for the impacts EWEs had on their neighbor's lives. Emphasis on relationships and systems of care and support are critical as they highlight the less-documented strengths of women living in informal settlements; in areas so often characterized by deficit, acknowledgment and further exploration of strength and resilience is paramount. EWE's negative impact on women's social lives, such as isolation or lack of privacy, warrant stakeholders' attention as it may interrupt this vital source of strength. While social impacts were cited less frequently than other areas of impact, more exploration is needed to understand how women's social relationships may be affected by EWEs and the protective nature of women's social supports amidst a changing climate and threats to health and wellbeing.

Our findings suggest that the impacts women experience intersect and overlap within and across systems. While pathways are not explicitly explored in this study, many women reported more than one impact at a time and discussed how these impacts co-occur or transact. For example, property damage may impact financial wellbeing, environmental dust may present a respiratory risk, and flooding that mobilizes trash and/or raw sewage and brings it into houses or businesses may present risks to both financial and physical health. Indeed, an impact in one of the levels described above has the potential to reverberate throughout the individuals' entire socio-ecological system. Amidst these intersecting impacts, we find potential for additional vulnerability (i.e., women who are struggling with their health while also navigating damage to their property and concerns with their job/business) but also strength and resilience (i.e., women whose businesses improved as a result of certain EWEs, which allowed them to save funds that help them to make repairs to their home). Resilience research tells us that examining these risk and protective factors is critical in understanding how best to support women as they continue to meet their and their family needs (40, 59–61) despite social and political marginalization and disinvestment systems that make it challenging for them to do so.

Our findings reveal the critical importance of the biosystem. Women identified some of the most common impacts that are the same as the description of the event itself (hotter-than-usual temperatures, colder-than-usual temperatures, bad air quality, and many mosquitos). Additionally, complex impacts involving physical environmental impacts co-occurring or causing additional financial, social, health, or environmental impacts at various levels of the ecology highlighted the critical importance of acknowledging the human-environment interface. We struggled with how to code these responses within the confines of Bronfenbrenner's ecological systems theory, but the addition of the biosystem to ecological systems theory (39, 40) helps bring life and understanding to these findings. The biosystem creates space for the experience of the event itself to have an impact and for us to better understand the impacts in the physical environment that co-occur or cause other social, economic, environmental, or health impacts at different levels of the ecology. For example, extreme heat experienced individually, within the body, or collectively, within the broader environment is impactful. Our bodies and communities, as embedded systems within the wider natural environment or climate, physically and intuitively experience changes in the natural system, especially extreme events, as an impact whether or not they are accompanied by other symptoms or impacts (e.g., being too hot or too cold is an impact).

Our findings also highlight the critical importance of the chronosystem in climate change-related research. The chronosystem provides an important context for understanding women's experience of EWE impacts. The impact of EWEs is likely to be experienced differently when coming at a time when they do not usually occur. Note that the language we use for heat and cold events is “hotter than usual” or “colder than usual,” which acknowledges that these events may be lasting longer than usual or are happening at unexpected times. While heavy rains, flooding, and drought are likely to be impactful at any time due to the previously mentioned vulnerabilities of informal settlements, the timing of the events—either lasting longer than they usually do or occurring at unexpected times—impact women's daily lives in a variety of ways. As climate change affects the frequency and intensity of events, seasons shift, and with them, our expectations of what is coming and our ability to prepare. This is particularly true in places like East Africa. East Africa (EA) is one of the most vulnerable regions of Africa (62) to climate change-related extreme weather events (EWEs), with studies showing increasing trends in temperature extremes and, to a lesser extent, changes to seasonal and annual rainfall (63). The chronosystem helps us understand impact data in its historical context of climate change, noting how the impacts have continued to escalate over time and are expected to change. Further, this exacerbation of climate change impacts is intertwined with historical legacies of political and social marginalization and government disinvestment in East Africa, which not only heighten the vulnerabilities of informal settlements but also limit their capacity for resilience and adaptation, making the consequences of these events far-reaching and systemic (64). Given the severity of the impacts women are already experiencing in informal settlement communities, there is urgency in acting now to increase informal settlement's abilities to adapt appropriately to climate change.

If the global community is to address the consequences of climate change on those most vulnerable, we must seek to prioritize research focused on the impacts on and co-creation of adaptation policies and interventions with community members themselves (65). The perspectives of women participating in this study presented a small glimpse into the complex, multidimensional, and interconnected impacts climate change and related EWEs may have on their physical and mental health, financial wellbeing, social relationships, built environment, and the surrounding physical environment. Stakeholders can utilize these findings to identify risk and protective factors, points for intervention, and areas for future exploration. As the global community learns more about the impacts of climate change on our world, we must turn to members of the communities most vulnerable to their effects—to help us understand their impacts and implications, identify areas of strength, and work collaboratively to develop solutions that mitigate harm.

While this study adds to existing research focused on the impacts of climate change and related EWEs on highly climate-vulnerable populations (women living in informal settlements), it was not without limitations. First, our findings come from brief text responses embedded in a structured survey. Hence, the responses were short, and there were no opportunities for probing or additional follow-up questions based on women's responses. More in-depth discussions on these topics are needed. Second, verbatim responses were entered into text boxes embedded into the digital surveys by our community data collectors. Autocorrect and typos made some of the responses challenging to interpret. Finally, the findings and our interpretations should consider possible bias related to the positionality and approaches of the study team (66). Study authors include members of the Kenyan research team, including the field manager and a member of the data collection team from Kibera, masters, doctoral students, and faculty from several US universities who represent a range of racial, national, and gender identities. Although the research team makes efforts to include community members, many authors, except for one team member who is a resident of Kibera and Mathare, are outsiders to the communities in which data were collected. To account for bias, researchers worked closely with the team of 16 women living in Mathare and Kibera development to review, edit, collect data, and translate findings throughout the study.

## Conclusion

Our findings complement the existing literature focused on the impacts of EWEs in informal settlements and add important context to the intersecting environmental, economic, and social impacts that women living in informal settlements in Nairobi, Kenya experience. Our findings reveal the complex, co-occurring, and interconnected nature of the impacts of climate change related to EWEs for women living in informal settlements, highlighting the need for holistic and comprehensive approaches to exploring and mitigating the impacts of climate change. Finally, our findings highlight the critical importance of the chrono- and biosphere systems in research focused on the impacts of climate change and related EWEs among climate-vulnerable communities and marginalized populations within them. Overall, we advocate for future exploration, intervention, and adaptation that center the experiences of and co-create with communities/populations expected to be most vulnerable to climate change, such as women in informal settlements.

## Data availability statement

The raw data supporting the conclusions of this article will be made available by the authors, without undue reservation.

## Ethics statement

The studies involving humans were approved by the Columbia University Internal Review Board and The Kenya Medical Research Institute (KEMRI). The studies were conducted in accordance with the local legislation and institutional requirements. The participants provided their written informed consent to participate in this study.

## Author contributions

AB: Conceptualization, Formal analysis, Writing – original draft, Writing – review & editing. SO: Conceptualization, Data curation, Formal analysis, Writing – review & editing. MD: Conceptualization, Data curation, Formal analysis, Project administration, Writing – review & editing. LP: Conceptualization, Formal analysis, Writing – review & editing. EU: Conceptualization, Writing – review & editing. LO: Conceptualization, Writing – review & editing. HB: Writing – review & editing. CM: Conceptualization, Writing – review & editing. CL: Conceptualization, Writing – review & editing. LY: Conceptualization, Writing – review & editing. SSW: Conceptualization, Formal analysis, Supervision, Writing – original draft, Writing – review & editing. SCW: Conceptualization, Formal analysis, Funding acquisition, Methodology, Project administration, Supervision, Writing – original draft, Writing – review & editing.

## References

[1] World Health Organization. Climate Change and Health Fact Sheet. Geneva: WHO (2021).

[2] Darkey D Kariuki A. A study on quality of life in Mathare, Nairobi, Kenya. J Hum Ecol. (2013) 41:207–19. 10.1080/09709274.2013.1190656931627277

[3] Cianconi P Betrò S Janiri L. The impact of climate change on mental health: a systematic descriptive review. Front Psychiatry. (2020) 11:490206. 10.3389/fpsyt.2020.00074 32210846 PMC7068211

[4] Dhimal M Bhandari D Dhimal ML Kafle N Pyakurel P Mahotra N. Impact of climate change on health and well-being of people in Hindu Kush Himalayan region: a narrative review. Front Physiol. (2021) 12:651189. 10.3389/fphys.2021.651189 34421631 PMC8378503

[5] Norris FH Friedman MJ Watson PJ Byrne CM Diaz E Kaniasty K. 60,000 disaster victims speak: part I. An empirical review of the empirical literature, 1981-−2001. Psychiatry. (2002) 65:207–39. 10.1521/psyc.65.3.207.20173 12405079

[6] Obradovich N Migliorini R Paulus MP Rahwan I. Empirical evidence of mental health risks posed by climate change. Proc Nat Acad Sci. (2018) 115:10953–8. 10.1073/pnas.1801528115 30297424 PMC6205461

[7] World Health Organization. Gender, Climate Change and Health. Geneva: World Health Organization (2014).

[8] Otieno PO Wambiya EO Mohamed SM Mutua MK Kibe PM Mwangi B. Access to primary healthcare services and associated factors in urban slums in Nairobi-Kenya. BMC Public Health. (2020) 20:1–9. 10.1186/s12889-020-09106-5 32571277 PMC7310125

[9] Oxfam Women's Empowerment National National Organization of Peer and Site Enterpriss Promotion. Gender and Power Analysis in Five Urban Informal Settlements – Nairobi, Kenya. Nairobi (2015).

[10] UN-HABITAT. State of the World's Cities 2006/7. New York, NY (2006).31207858

[11] Government of Kenya. National Slum Upgrading and Prevention Policy. Nairobi: Ministry of Land, Housing, and Urban Development (2016).

[12] UN-HABITAT. Slum Almanac 2015–2016: Tracking Improvement in the Lives of Slum Dwellers. Nairobi: United Nations Human Settlements Programme (UN-Habitat) (2015).

[13] Thorn J Thornton TF Helfgott A. Autonomous adaptation to global environmental change in peri-urban settlements: evidence of a growing culture of innovation and revitalisation in Mathare Valley Slums, Nairobi. Global Environ Change. (2015) 31:121–31. 10.1016/j.gloenvcha.2014.12.009

[14] University of Cape Town. Nairobi Climate Profile: Full Technical Version. Cape Town: University of Cape Town (2017).

[15] Mulligan J Harper J Kipkemboi P Ngobi B Collins A. Community-responsive adaptation to flooding in Kibera, Kenya. Proc Inst Civil Eng Eng Sustain. (2016) 170:268–80. 10.1680/jensu.15.0006026962031

[16] Fox S. The political economy of slums: theory and evidence from Sub-Saharan Africa. World Dev. (2014) 54:191–203. 10.1016/j.worlddev.2013.08.00529747633

[17] Satterthwaite D Archer D Colenbrander S Dodman D Hardoy J Patel S. Responding to Climate Change in Cities and in their Informal Settlements and Economies. Edmonton: International Institute for Environment and Development (2018), p. 61.

[18] APHRC. Population and Health Dynamics in Nairobi's Informal Settlements: Report of the Nairobi Cross-Sectional Slums Survey (NCSS) 2012. Nairobi: APHRC (2014).

[19] Winter SC Obara LM McMahon S. Intimate partner violence: a key correlate of women's physical and mental health in informal settlements in Nairobi, Kenya. PLoS ONE. (2020) 15:e0230894. 10.1371/journal.pone.0230894 32240207 PMC7117691

[20] Swart E. Gender-based violence in a kenyan slum: creating local, woman-centered interventions. J Soc Serv Res. (2012) 38:427–38. 10.1080/01488376.2012.676022

[21] Hambrecht E Tolhurst R Whittaker L. Climate change and health in informal settlements: a narrative review of the health impacts of extreme weather events. Environ Urban. (2022) 34:122–50. 10.1177/09562478221083896

[22] Chant S McIlwaine C. Cities, Slums and Gender in the Global South: Towards a Feminised Urban Future. London: Routledge (2016). 10.4324/9781315862996

[23] Corburn J Ngau P Karanja I Makau J. Mathare Zonal Plan, Nairobi, Kenya: Collaborative Plan for Informal Settlement Upgrading. Berkeley: University of California (2012).

[24] Suda C. Gender disparities in the Kenyan labour market: implications for poverty reduction. Nord J Afr Stud. (2002) 11:21. 10.53228/njas.v11i3.344

[25] Kyobutungi C Ziraba AK Ezeh A Yé Y. The burden of disease profile of residents of Nairobi's slums: results from a Demographic Surveillance System. Popul Health Metr. (2008) 6:1–8. 10.1186/1478-7954-6-1 18331630 PMC2292687

[26] Bronfenbrenner U. The Ecology of Human Development: Experiments by Nature and Design. Cambridge: Harvard University Press. (1979). 10.4159/9780674028845

[27] Crandon TJ Scott JG Charlson FJ Thomas HJ. A social–ecological perspective on climate anxiety in children and adolescents. Nat Clim Chang. (2022) 12:123–31. 10.1038/s41558-021-01251-y

[28] Mutisya E Yarime M. Understanding the grassroots dynamics of slums in Nairobi: the dilemma of Kibera informal settlements. Int Trans J Eng Manag Appl Sci Technol. (2011) 2:197–213.

[29] Scott AA Misiani H Okoth J Jordan A Gohlke J Ouma G. Temperature and heat in informal settlements in Nairobi. PLoS ONE. (2017) 12:e0187300. 10.1371/journal.pone.0187300 29107977 PMC5673164

[30] Douglas I. The challenge of urban poverty for the use of green infrastructure on floodplains and wetlands to reduce flood impacts in intertropical Africa. Landsc Urban Plan. (2018) 180:262–72. 10.1016/j.landurbplan.2016.09.025

[31] OpenStreetMap Contributors. Nairobi, Kenya [Map]. (2022). Available online at: https://www.openstreetmap.org/ (accessed April 10, 2024).

[32] Planet Labs PBC. Planet Application Program Interface: In Space for Life on Earth. Planet (2018). Available online at: https://api.planet.com (accessed April 15, 2024).

[33] Humanitarian Data Exchange (HDX). OpenStreetMap Data - Kenya Waterways [Dataset]. (2024). Available online at: https://data.humdata.org/dataset/hotosm_ken_waterways (accessed April 10, 2024).

[34] Winter S Barchi F Dzombo MN. Drivers of women's sanitation practices in informal settlements in sub-Saharan Africa: a qualitative study in Mathare Valley, Kenya. Int J Environ Health Res. (2018) 28:609–25. 10.1080/09603123.2018.1497778 30027750

[35] Lind K Oldendick R. Comparison of the accuracy of the last birthday versus the next birthday methods for random selection of household respondents. Age. (2000) 231:e7.

[36] World Health Organization. Putting Women First: Ethical and safety Recommendations for Research on Domestic Violence against Women. Geneva: Department of Gender Women and Health (2001).

[37] Seneviratne SI Zhang X Adnan M Badi W Dereczynski C Di Luca A. Weather and climate extreme events in a changing climate. In: Climate Change 2021: The Physical Science Basis. Contribution of Working Group I to the Sixth Assessment Report of the Intergovernmental Panel on Climate Change. Cambridge (2021).

[38] Patton MQ. Qualitative Research and Evaluation Methods. London: Sage (2002).

[39] Elliott S Davis JM. Challenging taken-for-granted ideas in early childhood education: a critique of Bronfenbrenner's ecological systems theory in the age of post-humanism. In:Cutter-Mackenzie-Knowles A Malone K Barratt Hacking E, editors. Research Handbook on Childhoodnature: Assemblages of Childhood and Nature Research. Cham: Springer (2020), p. 1119–54. 10.1007/978-3-319-67286-1_60

[40] Folke C Biggs R Norström AV Reyers B Rockström J. Social-ecological resilience and biosphere-based sustainability science. Ecol Soc. (2016) 21:art41. 10.5751/ES-08748-21034130174746

[41] Gasparrini A Guo Y Hashizume M Lavigne E Zanobetti A Schwartz J. Mortality risk attributable to high and low ambient temperature: a multicountry observational study. Lancet. (2015) 386:369–75. 10.1016/S0140-6736(14)62114-0 26003380 PMC4521077

[42] Li M Ferreira S Smith TA. Temperature and self-reported mental health in the United States. PLoS ONE. (2020) 15:e0230316. 10.1371/journal.pone.0230316 32210473 PMC7094821

[43] Xu Z Etzel RA Su H Huang C Guo Y Tong S. Impact of ambient temperature on children's health: a systematic review. Environ Res. (2012) 117:120–31. 10.1016/j.envres.2012.07.002 22831555

[44] Egondi T Kyobutungi C Kovats S Muindi K Ettarh R Rocklöv J. Time-series analysis of weather and mortality patterns in Nairobi's informal settlements. Glob Health Action. (2012) 5:19065. 10.3402/gha.v5i0.19065 23195509 PMC3509073

[45] Egondi T Kyobutungi C Rocklöv J. Temperature variation and heat wave and cold spell impacts on years of life lost among the urban poor population of Nairobi, Kenya. Int J Environ Res Public Health. (2015) 12:2735–48. 10.3390/ijerph120302735 25739007 PMC4377929

[46] Moncada E. The politics of urban violence: challenges for development in the global south. Stud Comp Int Dev. (2013) 48:217–39. 10.1007/s12116-013-9133-z

[47] Salahub JE Gottsbacher M De Boer J. Social Theories of Urban Violence in the Global South: Towards Safe and Inclusive Cities. London: Routledge (2018). 10.4324/9781351254724

[48] Ezeh A Oyebode O Satterthwaite D Chen Y-F Ndugwa R Sartori J. The history, geography, and sociology of slums and the health problems of people who live in slums. Lancet. (2017) 389:547–58. 10.1016/S0140-6736(16)31650-6 27760703

[49] Lilford RJ Oyebode O Satterthwaite D Melendez-Torres GJ Chen Y-F Mberu B. Improving the health and welfare of people who live in slums. Lancet. (2017) 389:559–70. 10.1016/S0140-6736(16)31848-7 27760702

[50] Kienja K. Pollution of Urban Waterways in Nairobi: A Case Study of Mathare 4B Village, Nairobi, Kenya. Christchurch: University of Canterbury (2017).

[51] Mburu SW. Relationship between socio-economic exclusion and community based Waste management practices in Kibera informal settlements, Nairobi County (Ph.D Thesis). Kenyatta University (2017).

[52] Olale PO. Implications of Land Tenure Security on Sustainable Land Use in Informal Settlements in Nairobi. Kenya: University of Nairobi (2015).

[53] Corburn J Njoroge P Weru J Musya M. Urban climate justice, human health, and citizen science in Nairobi's informal settlements. Urban Sci. (2022) 6:36. 10.3390/urbansci6020036

[54] World Bank. Fact sheet: An Adjustment to Global Poverty Lines (2022). Available online at: https://www.worldbank.org/en/news/factsheet/2022/05/02/fact-sheet-an-adjustment-to-global-poverty-lines (accessed April 20, 2024).

[55] Celentano G Habert G. Beyond materials: the construction process in space, time and culture in the informal settlement of Mathare, Nairobi. Dev Eng. (2021) 6:100071. 10.1016/j.deveng.2021.100071

[56] Winter SC Sommer M Obara LM Nair D. “There is no place to dispose them. What would you have me do?”: a qualitative study of menstruation in the unique physical and social environment in informal settlements in Nairobi, Kenya. Health Place. (2022) 78:102932. 10.1016/j.healthplace.2022.102932 36370631

[57] Amuyunzu-Nyamongo M Ezeh AC. A qualitative assessment of support mechanisms in informal settlements of Nairobi, Kenya. J Poverty. (2005) 9:89–107. 10.1300/J134v09n03_05

[58] Morgner C Ambole A Anditi C Githira D. Exploring the dynamics of social networks in urban informal settlements: the case of Mathare Valley, Kenya. Urban Forum. (2020) 31:489–512. 10.1007/s12132-020-09389-2

[59] Gaisie E Adu-Gyamfi A Owusu-Ansah JK. Gender and household resilience to flooding in informal settlements in Accra, Ghana. J Environ Plann Manag. (2022) 65:1390–413. 10.1080/09640568.2021.1930522

[60] Mitra S Mulligan J Schilling J Harper J Vivekananda J Krause L. Developing risk or resilience? Effects of slum upgrading on the social contract and social cohesion in Kibera, Nairobi. Environ Urban. (2017) 29:103–22. 10.1177/0956247816689218

[61] Muhanguzi FK Boonabaana B Sanya LN Kavuma SN Kyomuhendo GB Ludgate N. The meanings of resilience in climate justice: women smallholder farmers' responses to agricultural shocks in Uganda under the spotlight. Agenda. (2023) 37:106–23. 10.1080/10130950.2023.2245844

[62] Gebrechorkos SH Hülsmann S Bernhofer C. Changes in temperature and precipitation extremes in Ethiopia, Kenya, and Tanzania. Int J Climatol. (2019) 39:18–30. 10.1002/joc.5777

[63] Gebrechorkos SH Hülsmann S Bernhofer C. Long-term trends in rainfall and temperature using high-resolution climate datasets in East Africa. Sci Rep. (2019) 9:11376. 10.1038/s41598-019-47933-8 31388068 PMC6684806

[64] James N. The effects of climate change on informal settlements-Commentary by Nokwenama James, 31 January 2023. Town Reg Plann. (2023) 82:1–3. 10.38140/trp.v82i.661638746564

[65] Winter SC Winter MR Plaxico L Balakrishnan AK Dzombo M Tabb LP. Extreme weather should be defined according to impacts on climate-vulnerable communities. Nat Clim Change. (2024) 14:462–7. 10.1038/s41558-024-01983-7 PMC12360233 40831698

[66] Jafar AJ. What is positionality and should it be expressed in quantitative studies? Emerg Med J. (2018) 35:323–4. 10.1136/emermed-2017-207158 29326239

